# Sorbitol reduction via govorestat ameliorates synaptic dysfunction and neurodegeneration in sorbitol dehydrogenase deficiency

**DOI:** 10.1172/jci.insight.164954

**Published:** 2023-05-22

**Authors:** Yi Zhu, Amanda G. Lobato, Adriana P. Rebelo, Tijana Canic, Natalie Ortiz-Vega, Xianzun Tao, Sheyum Syed, Christopher Yanick, Mario Saporta, Michael Shy, Riccardo Perfetti, Shoshana Shendelman, Stephan Züchner, R. Grace Zhai

**Affiliations:** 1 Department of Molecular and Cellular Pharmacology,; 2 Graduate Program in Human Genetics and Genomics,; 3 Dr. John T. Macdonald Foundation Department of Human Genetics and John P. Hussman Institute for Human Genomics, University of Miami Miller School of Medicine, Miami, Florida, USA.; 4 Department of Physics, University of Miami, Coral Gables, Florida, USA.; 5 Graduate Program in Cellular and Molecular Pharmacology,; 6 Department of Neurology, and; 7 Graduate Program in Neuroscience, University of Miami Miller School of Medicine, Miami, Florida, USA.; 8 Department of Neurology, Carver College of Medicine, University of Iowa, Iowa City, Iowa, USA.; 9 Research & Development, Applied Therapeutics, New York, New York, USA.

**Keywords:** Cell Biology, Neuroscience, Genetic diseases, Neurodegeneration, Neurological disorders

## Abstract

Sorbitol dehydrogenase (SORD) deficiency has been identified as the most frequent autosomal recessive form of hereditary neuropathy. Loss of SORD causes high sorbitol levels in tissues due to the inability to convert sorbitol to fructose in the 2-step polyol pathway, leading to degenerative neuropathy. The underlying mechanisms of sorbitol-induced degeneration have not been fully elucidated, and no current FDA-approved therapeutic options are available to reduce sorbitol levels in the nervous system. Here, in a *Drosophila* model of SORD deficiency, we showed synaptic degeneration in the brain, neurotransmission defect, locomotor impairment, and structural abnormalities in the neuromuscular junctions. In addition, we found reduced ATP production in the brain and ROS accumulation in the CNS and muscle, indicating mitochondrial dysfunction. Applied Therapeutics has developed a CNS-penetrant next-generation aldose reductase inhibitor (ARI), AT-007 (govorestat), which inhibits the conversion of glucose to sorbitol. AT-007 significantly reduced sorbitol levels in patient-derived fibroblasts, induced pluripotent stem cell–derived (iPSC-derived) motor neurons, and *Drosophila* brains. AT-007 feeding in Sord-deficient *Drosophila* mitigated synaptic degeneration and significantly improved synaptic transduction, locomotor activity, and mitochondrial function. Moreover, AT-007 treatment significantly reduced ROS accumulation in *Drosophila* CNS, muscle, and patient-derived fibroblasts. These findings uncover the molecular and cellular pathophysiology of SORD neuropathy and provide a potential treatment strategy for patients with SORD deficiency.

## Graphical abstract

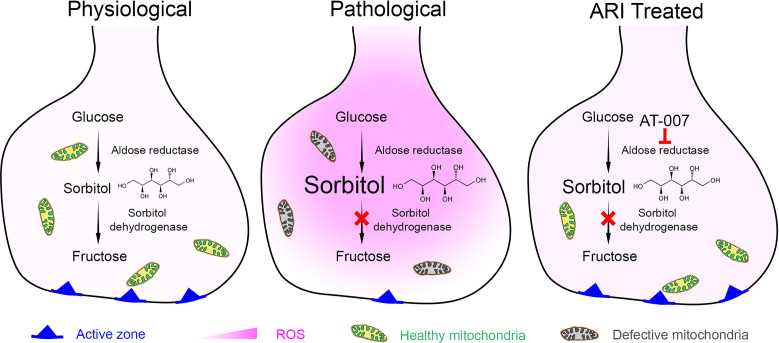

## Introduction

Sorbitol dehydrogenase (SORD) deficiency has been identified as the most frequent autosomal recessive form of hereditary neuropathy, affecting roughly 1 per 100,000 individuals (1). *SORD* mutations result in decreased levels and loss of function of the SORD enzyme, leading to neuronal sorbitol accumulation (2). The polyol pathway consists of 2 enzymes: aldose reductase (AR), which catalyzes the reduced nicotinamide adenine dinucleotide phosphate–mediated (NADPH-mediated) reduction of glucose to sorbitol, and SORD, which catalyzes the nicotinamide adenine dinucleotide^^+^^–mediated (NAD^^+^^-mediated) oxidation of sorbitol to fructose (3, 4). The net result of the polyol pathway is the formation of fructose from glucose and the transfer of reducing equivalents from NADPH to NAD^^+^^ (5–8). Loss of SORD causes high intracellular sorbitol levels due to the inability to convert sorbitol to fructose (9).

We have previously characterized the neuropathological and behavioral changes in a *Drosophila* model of SORD deficiency, including synaptic degeneration and progressive motor impairment (1). However, the mechanism of neuronal damage caused by sorbitol accumulation has not been fully elucidated. Several prior biochemical observations have suggested that increased polyol-associated nerve damage is associated with decreased nerve myo-inositol levels, decreased nerve conduction velocities, and decreased cholinergic acetyltransferase transport (3). Here, we focus on the molecular and cellular mechanism underlying sorbitol-induced neuronal toxicity. Based on our Sord-deficient *Drosophila* model, we characterized synaptic changes in the brain, assessed locomotor output using a newly developed geotaxis behavior platform, and analyzed the morphological changes in neuromuscular junctions (NMJs). We investigated mitochondrial pathology by staining mitochondrial markers, quantifying neuronal ATP content, and assessing ROS accumulation. Collaborating with Applied Therapeutics, we tested a next-generation CNS-penetrant AR inhibitor (ARI), AT-007 (govorestat), by treating patient-derived fibroblasts and induced pluripotent stem cell–derived (iPSC-derived) motor neurons as well as by feeding Sord-deficient flies. Our study revealed mitochondrial dysfunction as a critical contributor to sorbitol-induced neuropathy and provided a therapeutic solution to reduce intracellular sorbitol levels and protect against sorbitol-induced neurotoxicity.

## Results

### Development of a next-generation ARI AT-007.

**Figure 1:**
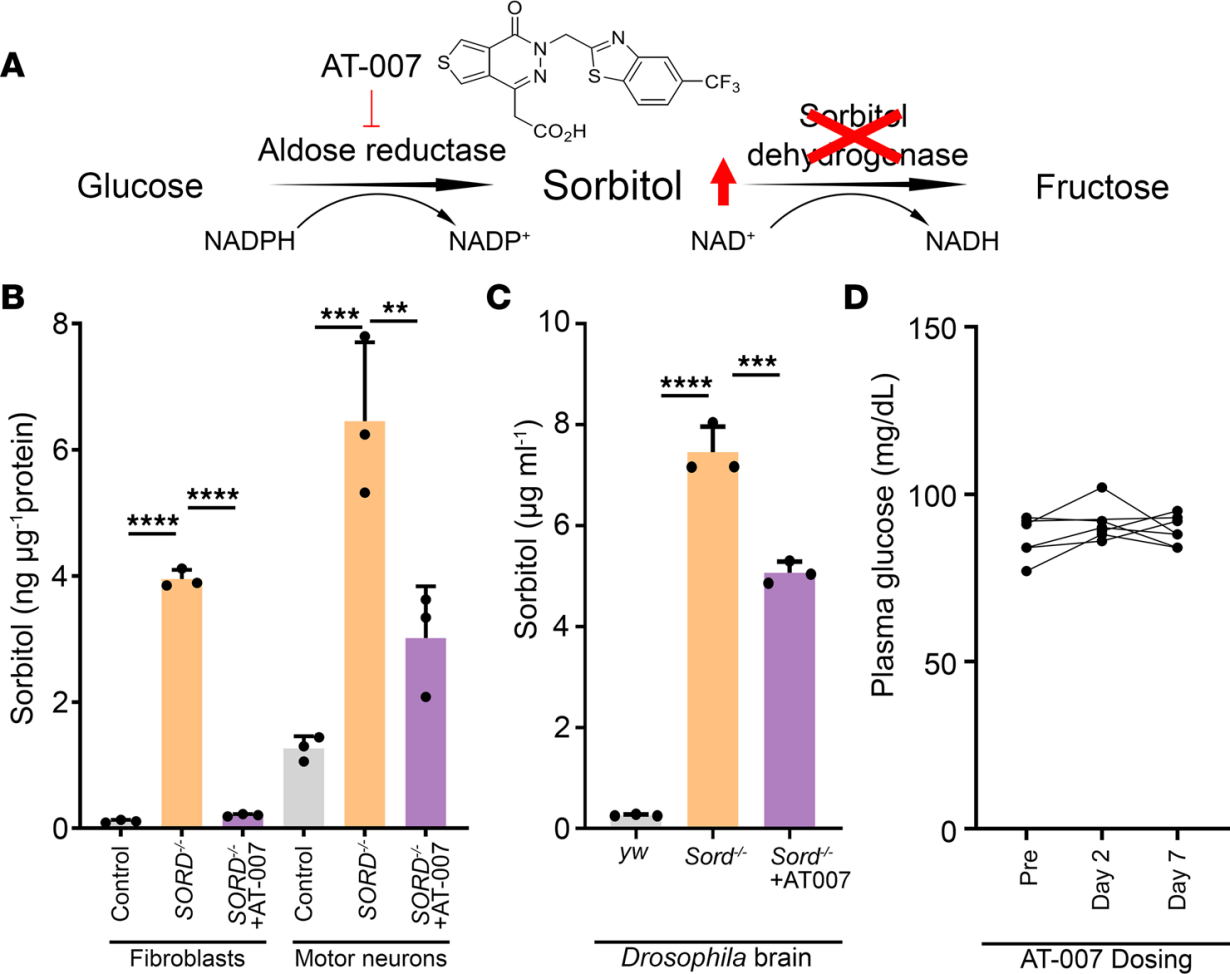
Aldose reductase inhibitor AT-007 significantly reduced sorbitol levels in vitro and in vivo. (**A**) A schematic graph showing the 2-step polyol pathway converting glucose to sorbitol by aldose reductase (AR) and converting sorbitol to fructose by sorbitol dehydrogenase (SORD). SORD deficiency causes an increase in sorbitol levels. AT-007 (govorestat) is a potentially novel ARI developed by Applied Therapeutics that potently inhibits AR, thereby reducing intracellular sorbitol accumulation. (**B**) Sorbitol levels in fibroblasts or motor neurons derived from SORD-deficient patients or healthy controls. (**C**) Sorbitol levels in brains of *yw* flies (control) fed with DMSO and Sord-deficient flies (*Sord2^MB01265/MB01265^*) fed with DMSO or 10 μg/mL AT-007 10 DAE. One-way ANOVA was performed for statistical analysis. Data are presented as mean ± SD, *n* = 3, **P < 0.01, ***P < 0.001, ****P < 0.0001. (**D**) Plasma glucose levels in patients with a SORD deficiency treated with AT-007 (data available for 7 of 8 patients treated).

SORD deficiency results in neuronal sorbitol accumulation (3). AR is encoded by 1 gene and has a known structure and kinetic properties (4). To reduce sorbitol accumulation by inhibiting the conversion from glucose, a next-generation ARI was developed by Applied Therapeutics, named AT-007 or govorestat (Figure 1A). AT-007 is a CNS penetrant, orally active ARI with IC__50__ of 100 pM. AT-007 has been shown to reduce toxic galactitol levels and prevent disease complications in galactose-1-phosphate uridyl transferase deficiency (10), and its effect on SORD deficiency is being evaluated in a phase III trial.

We evaluated the efficacy of AT-007 in reducing sorbitol levels in patient-derived fibroblasts, iPSC-derived motor neurons, and *Drosophila* brains (Figure 1, B and C). Sorbitol levels were significantly higher in patient-derived fibroblasts (3.95 ± 1.46 ng/μg protein) compared with that in control fibroblasts (0.11 ± 0.02 ng/μg protein) and were significantly reduced after AT-007 treatment (0.21 ± 0.02 ng/μg protein). Next, we measured sorbitol levels in iPSC-derived motor neurons. Notably, in control individuals, we found a higher baseline sorbitol level in motor neurons (1.27 ± 0.19 ng/μg protein) than in fibroblasts, indicating that motor neurons are more vulnerable to sorbitol accumulation. Sorbitol level was remarkably increased in motor neurons derived from SORD-deficient patients (6.46 ± 1.25 ng/μg protein), though it could be suppressed by AT-007 treatment (3.02 ± 0.82 ng/μg protein). Finally, we measured sorbitol levels in the *Drosophila* brain extracts. We found a significant increase in sorbitol levels in Sord-deficient fly brains (7,457 ng/mL) compared with that in control (*yw*) brains (260.7 ng/mL), though it could be reduced by 10 days of AT-007 (10 μg/mL) feeding (5,067 ng/mL). Taken together, our in vitro and in vivo models demonstrated that AT-007 is a robust ARI that significantly decreases sorbitol accumulation.

Since ARI inhibits the conversion of glucose to sorbitol, we investigated whether AT-007 treatment alters plasma glucose levels. Eight patients diagnosed with SORD deficiency were treated with AT-007 (20 mg/kg/day). Plasma glucose level was measured before the first dose, 2 days after the first dose, and after 7 days of consecutive dosing. No clinically significant changes in glucose levels after AT-007 treatment were detected (Figure 1D).

### AT-007 ameliorates synaptic degeneration in Sord-deficient flies.

**Figure 2:**
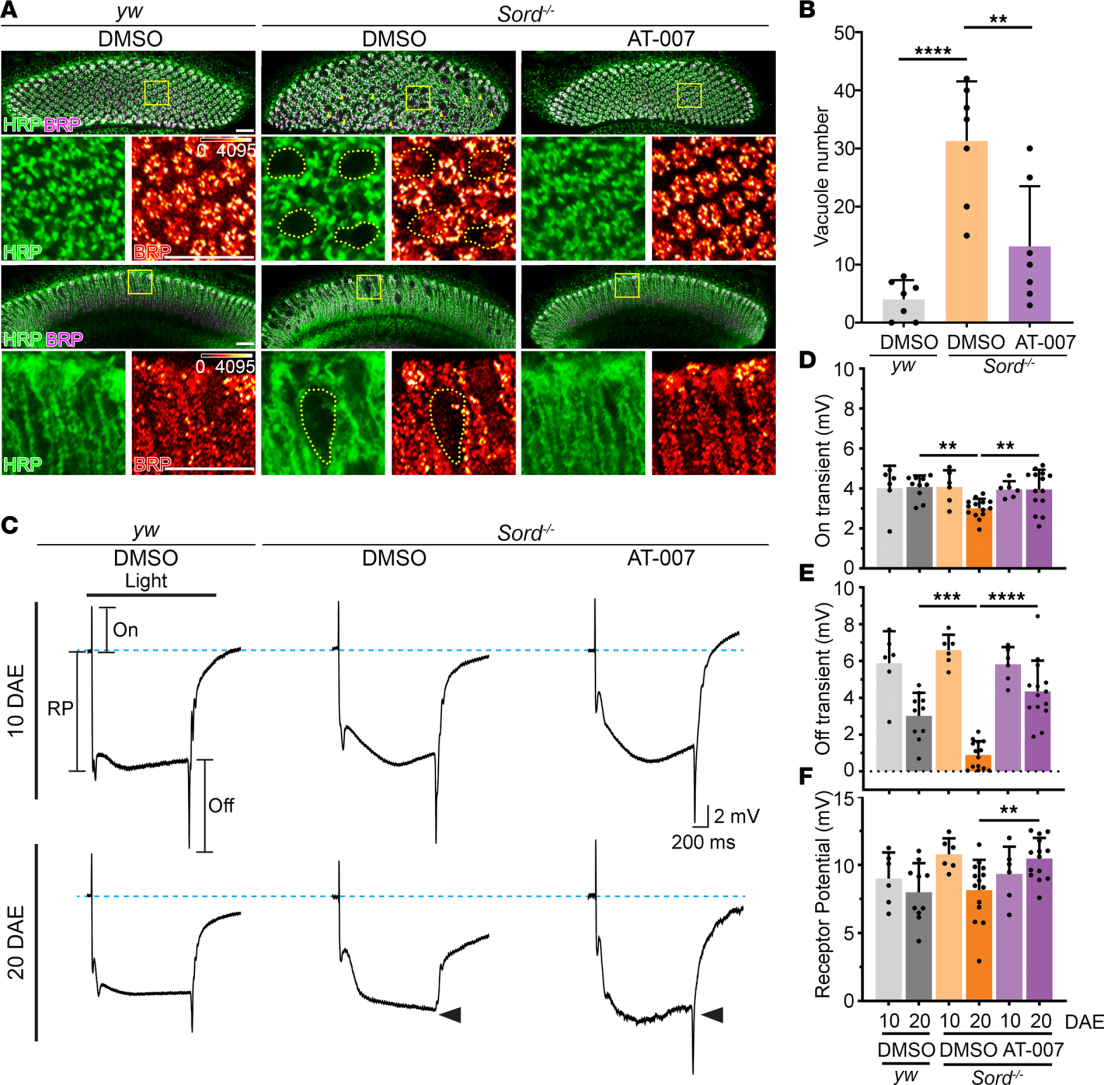
AT-007 ameliorates synaptic degeneration in Sord-deficient flies. (**A**) Brains of *yw* flies (control) fed with DMSO and Sord-deficient flies (*Sord2^MB01265/MB01265^*) fed with DMSO or 10 μg/mL AT-007 10 DAE were dissected and stained with HRP (green) or BRP (magenta). The upper panels show the cross-section of lamina cartridges, and the lower panels show the lamina longitudinal section highlighting the columnar photoreceptor axons. Yellow arrowheads indicate vacuole-like structures formed in the lamina regions. Boxed areas are shown in higher magnification. The fluorescence intensity of BRP is indicated using a heatmap. Scale bars: 15 μm. (**B**) Quantification of vacuole-like structures in the lamina. One-way ANOVA was performed for statistical analysis. Data are presented as mean ± SD, *n* = 7, **P < 0.01, ****P < 0.0001. (**C**) Electroretinogram analysis of *yw* flies (control) fed with DMSO and Sord-deficient flies fed with DMSO or 10 μg/mL AT-007 at 10 and 20 DAE. RP, receptor potential. (**D**–**F**) Quantification of On, Off, and RP amplitudes in **C**.

Defects in axonal transport and synaptic transmission have been implicated in many models of inherited neuropathies (11, 12). To evaluate axonal and synaptic integrity, we took advantage of the *Drosophila* visual system, where the photoreceptor neurons are highly organized in parallel columns and make synapses with lamina monopolar cells (13). We previously observed the age-dependent formation of vacuole-like structures in Sord-deficient flies by labeling neuronal membranes with horseradish peroxidase (HRP), indicating a progressive loss of synaptic terminals (1). We also reported a reduced level of synaptic active zone (AZ) marker Bruchpilot (BRP), indicating synaptic degeneration (1). Consistently, in this study, we observed a significant increase in vacuole-like structures in the lamina of Sord-deficient flies 10 days after eclosion (DAE) (Figure 2A). Feeding with AT-007 for 10 days significantly reduced the number of vacuole-like structures and maintained synaptic integrity in Sord-deficient flies (Figure 2, A and B).

To analyze the functions of photoreceptors, we performed electroretinogram (ERG) recordings to measure the electrical response to light stimulation (14) (Figure 2, C–F). At 10 DAE, in both control (*yw*) and Sord-deficient flies, we observed normal amplitudes of all ERG components, including On transient, Off transient, and receptor potential (RP). At 20 DAE, we observed a significant decrease in the On transient (Figure 2D) and almost a complete loss of the Off transient (Figure 2, C and E) in Sord-deficient flies, suggesting an age-dependent decline of synaptic transmission. Feeding with AT-007 for 20 days significantly improved On, Off, and RP components, indicating improved phototransduction and synaptic function.

### AT-007 improves the locomotor activity of Sord-deficient flies.

Motor weakness is a common symptom of peripheral neuropathy. The weakness can be mild or severe and may progress over time (15). In patients with SORD neuropathy, motor weakness is more prominent than sensory impairment. We previously reported that 59% (26 of 44) patients had distal upper limb weakness, and 98% had distal lower limb weakness (43 of 44), with disease severity ranging from mild to complete paralysis (1). The onset of the neuropathy in Sord-deficient patients occurred at an average age of 17 ± 8 years, and the most frequently reported issue at the start of the condition was difficulty walking (1). Locomotor function in *Drosophila* can be measured by the negative geotaxis behavior, which requires an intact neurocircuitry that senses gravity and conveys the resulting modulatory signal to downstream motor circuits to control muscle contractions (16). We have shown age-dependent decrease in locomotor activity in Sord-deficient flies in a traditional negative geotaxis assay in which we tapped down the flies to the bottom of the vials and counted the number of flies passing the 8 cm line in 10 seconds in the prior study. The earliest time point during which we observed a significantly reduced average passing rate was 40 DAE (1). However, this traditional method of negative geotaxis assay has several drawbacks; it is time-consuming and labor-intensive, involves inconsistent manual tapping force, and has a single output (average passing rate) with limited resolution to detect subtle locomotor changes (17).

**Figure 3:**
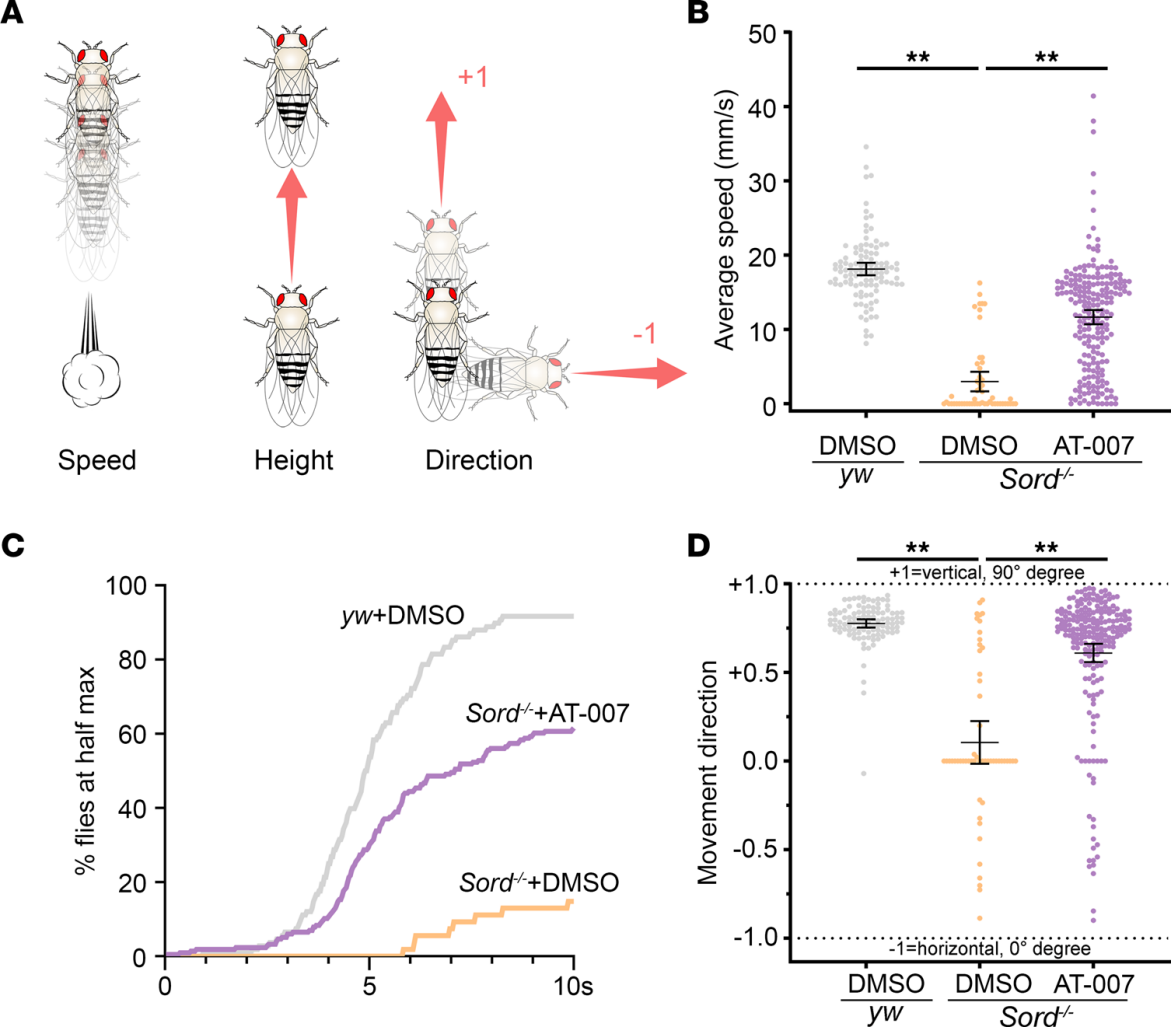
AT-007 improves the locomotor activity of Sord-deficient flies. (**A**) Fly geotactic activity was recorded using an automated monitoring system. The program automatically tracks fly locations and determines their speed, climbing rate, and movement direction in a 10-second time interval. As flies normally display an innate negative geotaxis response, movement direction was quantified between –1 (completely horizontal) to +1 (completely vertical). (**B**–**D**) Average movement speed (**B**), climbing rate (**C**), and movement direction (**D**) of *yw* flies (control) fed with DMSO (*n* = 12 flies ***×*** 9 trials + 6 flies ***×*** 10 trials = 168 tracks) and Sord-deficient flies (*Sord2^MB01265/MB01265^*) fed with DMSO (*n* = 12 flies ***×*** 10 trials + 6 flies ***×*** 9 trials = 174 tracks) or 10 μg/mL AT-007 (*n* = 24 flies ***×*** 9 trials = 216 tracks) at 10 DAE. One-way ANOVA was performed for statistical analysis. Data are presented as mean ± SEM, **P < 0.01 from trial-by-trial comparisons.

To address these limitations, we developed an automated geotaxis monitoring (AGM) system with computer-programmed motorized tapping and video tracking capabilities. A MatLab-based algorithm was developed to record and track the position of each fly individually at a resolution of 30 frames per second (fps) (Supplemental Videos 1 and 2; supplemental material available online with this article; https://doi.org/10.1172/jci.insight.164954DS1). By extracting information about speed, climbing rate, and movement direction (Figure 3A), we could dissect the affected component of locomotor circuitry upon sorbitol accumulation. Specifically, finding the correct movement direction requires proper gravity sensing, primarily the sensory component, whereas the movement speed and climbing rate depend on the integrity of the entire neurocircuitry of sensory and motor components (17).

We monitored the locomotor activity of flies within a 10-second window. As shown in an example video clip (Supplemental Video 3), compared with the control group (*yw*, vials 1 and 2), Sord-deficient flies (vials 3 and 4) exhibited reduced climbing speed (3.0 mm/s) compared with the control group (18.1 mm/s). Consistently, Sord-deficient flies showed a decreased climbing rate, as only about 20% of flies reached the half-max height (~7 cm) of the climbing tube, while the control group had an average passing rate of 90%. Feeding flies on 10 μg/mL AT-007 for 10 days (vials 5, 6, 7) significantly improved speed (11.7 mm/s) and climbing rate (average passing rate of 60%) (Figure 3, B and C). Next, we assessed the movement direction, with a score of +1.0 indicating complete vertical movement and a score of –1.0 indicating complete horizontal movement. We observed that movement in the control group was mostly vertical (direction score, 0.77). However, the direction score decreased to 0.10 in Sord-deficient flies, indicating impaired gravity sensing, and was improved to 0.61 by AT-007 feeding (Figure 3D). Collectively, our data demonstrate that Sord-deficient flies presented locomotor deficits, recapitulating the key clinical feature reported in patients with SORD deficiency–induced neuropathy (1). Our AGM analysis further revealed that the locomotor deficits involved defects primarily in mobility (speed and distance climbed) and movement direction and could be rescued by AT-007 feeding.

### Sord-deficient flies exhibit AZ structural abnormalities in the NMJs.

**Figure 4:**
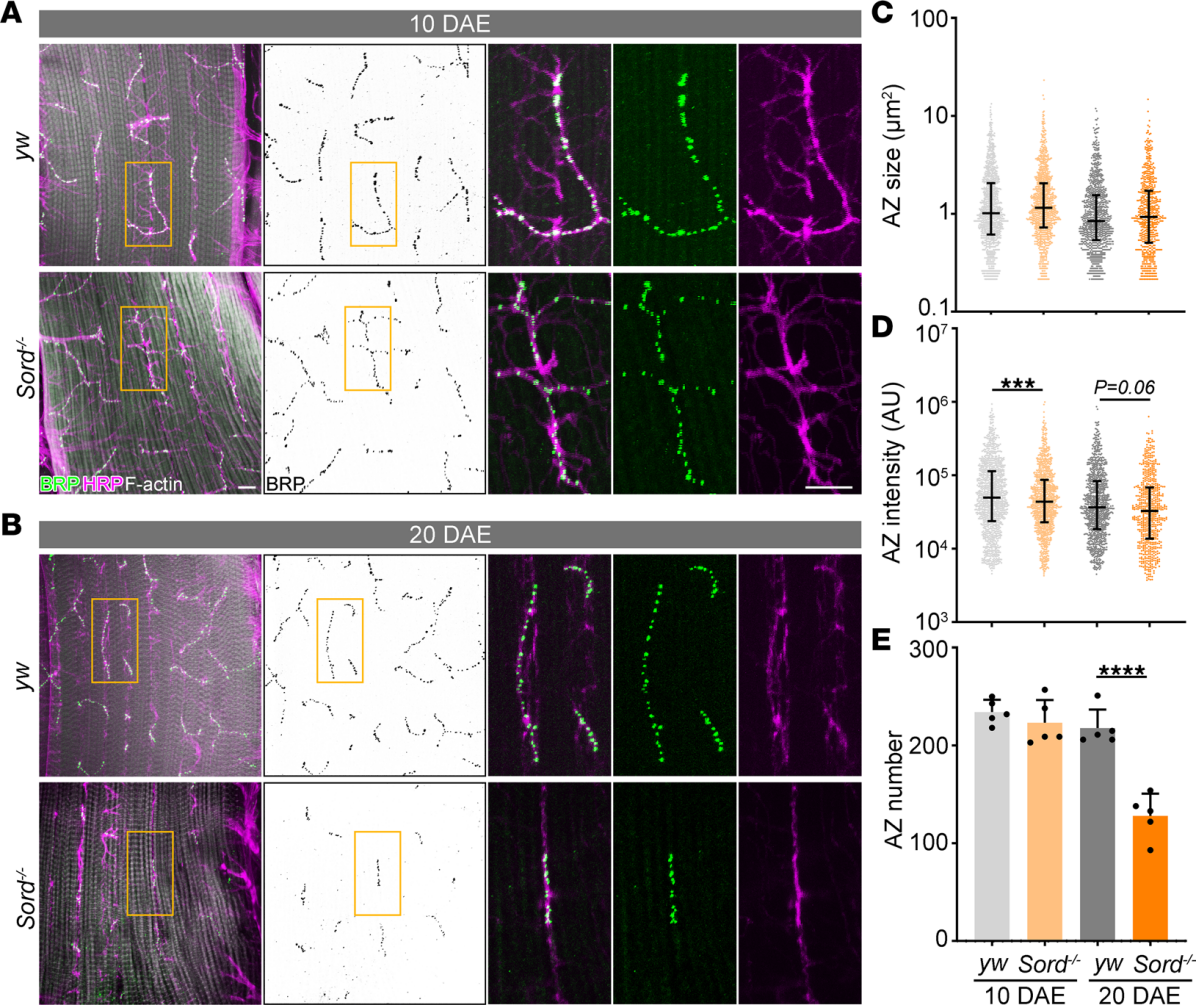
Sord-deficient flies exhibit active zone (AZ) structural abnormalities in the flight muscle neuromuscular junctions (NMJs). (**A** and **B**) Flight muscles of *yw* flies (control) and Sord-deficient flies were dissected at 10 and 20 DAE and stained with BRP, HRP, and phalloidin (F-actin). Boxed areas are shown in higher magnification. Scale bars: 10 μm. (**C**–**E**) Quantification of AZ size, intensity, and number in flight muscle NMJs. Data are presented as median ± interquartile range (**C** and **D**) and mean ± SD (**E**). ***P < 0.001, ****P < 0.0001. *n* = 5. Student’s *t* test was performed for statistical analysis.

**Figure 5:**
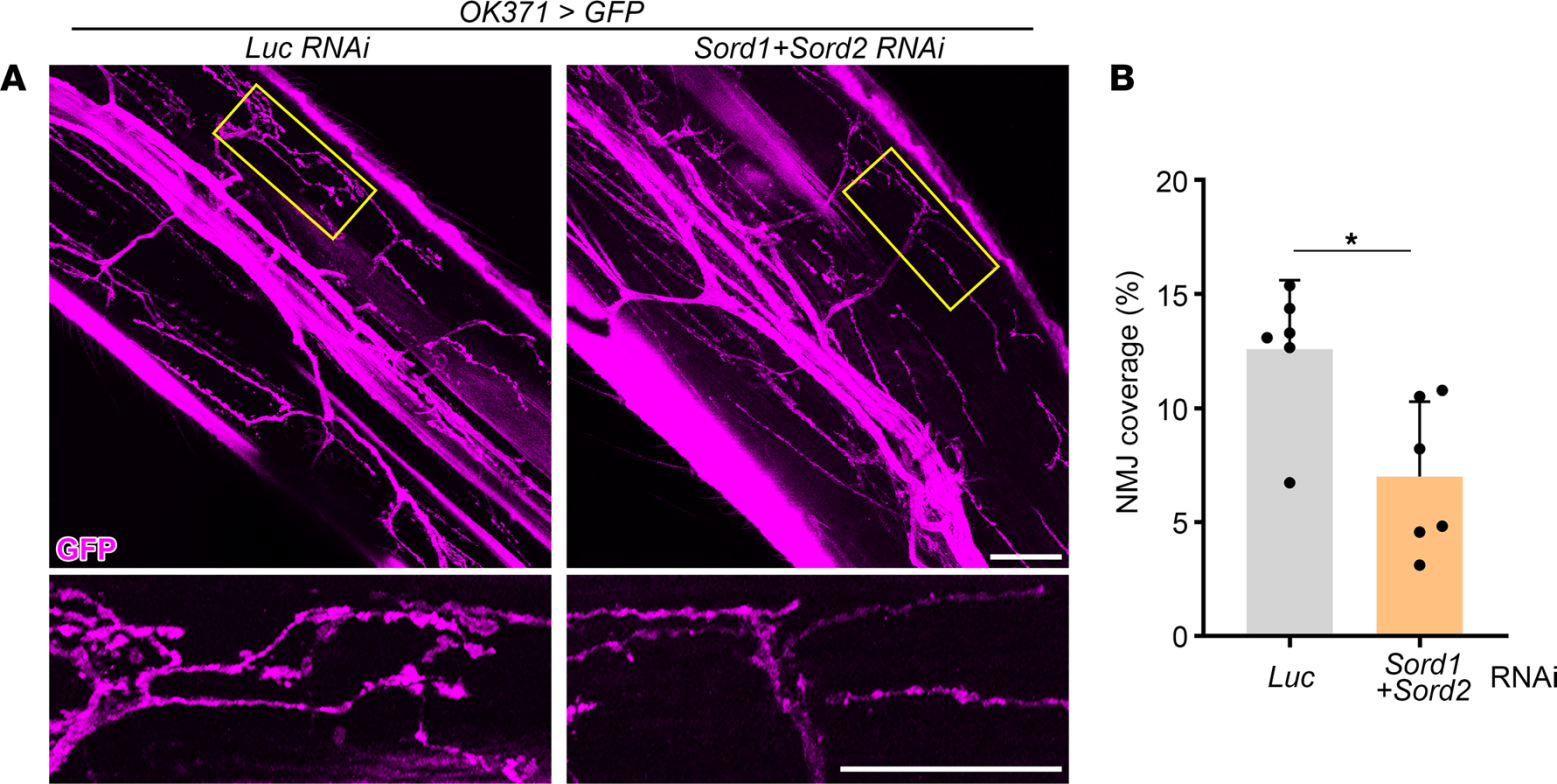
Sord-deficient flies exhibit reduced neuromuscular junctions (NMJ) coverage in the leg. (**A**) Legs of 5 DAE flies with motor neuron–specific knockdown of Sord1 and Sord2 were dissected and imaged. Legs of flies with motor neuron–specific knockdown of luciferase were used as controls. Leg motor neurons were labeled with GFP. Boxed NMJ areas are shown in higher magnification. Scale bars: 30 μm. (**B**) Quantification of NMJ coverage. In each group, 6 NMJs were chosen, and the percentage of areas covered by NMJ was quantified. Student’s *t* test was performed for statistical analysis. Data are presented as mean ± SD, *n* = 6, *P < 0.05.

The defect in locomotor activity suggests underlying compromised motor circuitry. To analyze synaptic structural changes in the motor system, we performed flight muscle dissections and examined the AZ integrity at NMJs. NMJ morphological defects have been reported in mammalian models of peripheral neuropathy but have not been investigated in SORD deficiency–induced neuropathy (18). Interestingly, we found an early-stage (10 DAE) AZ intensity reduction in Sord-deficient flies compared with control (*yw*) flies and a late-stage (20 DAE) AZ number reduction at NMJs, demonstrating a structural abnormality of the motor system (Figure 4). Next, we specifically knocked down Sord1 and Sord2 in the motor neurons and observed an age-dependent decrease in AZ size, intensity, and number at flight muscle NMJs, when compared with the control luciferase RNA interference (RNAi) (Supplemental Figure 1). Moreover, we labeled leg motor neurons using a GFP reporter and found a reduction in NMJ coverage in leg muscles (Figure 5). The morphological analyses of the NMJ showed synaptic and AZ structural abnormalities in the motor system that may contribute to locomotor impairment in Sord-deficient flies.

### Sord-deficient flies exhibit increased cleaved Caspase-3 activity.

**Figure 6:**
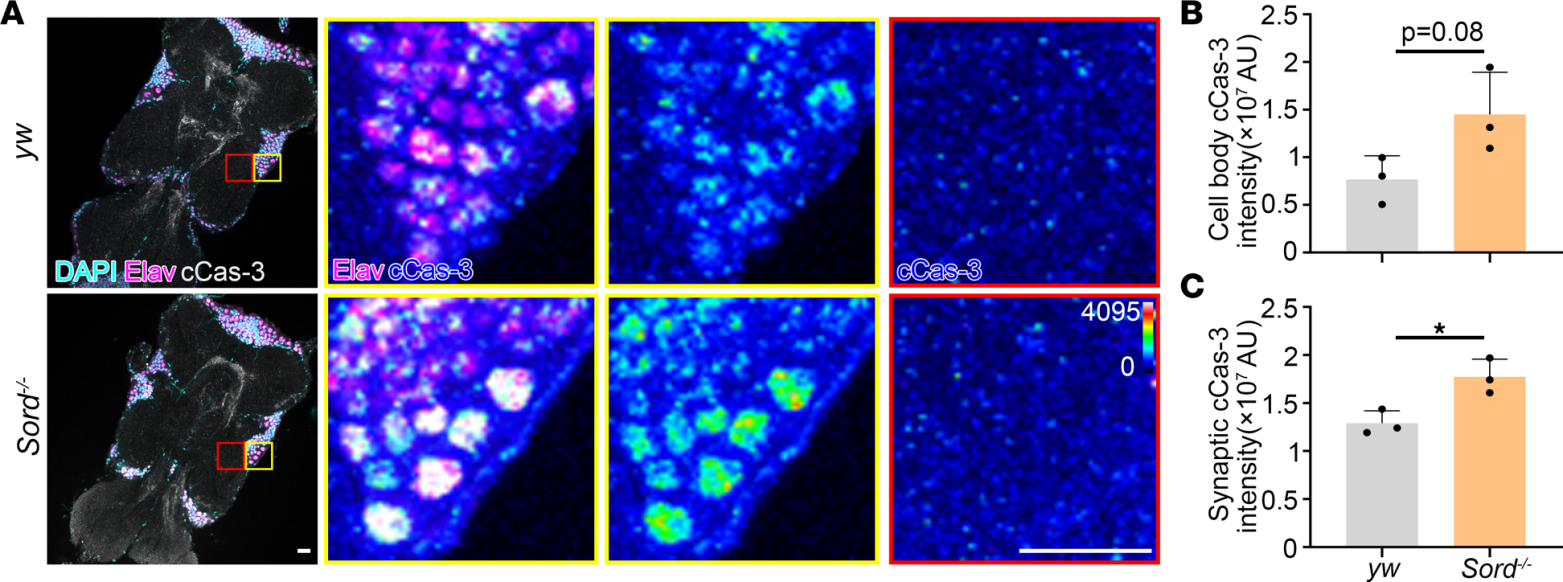
Sord-deficient flies show ventral nerve cord (VNC) vulnerability to apoptosis. (**A**) Ventral nerve cords of 10 DAE control *yw* and Sord-deficient flies were dissected and stained for DAPI (cyan), Elav (magenta), and cCas-3 (gray). Boxed areas are shown in higher magnification. The fluorescence intensity of cCas-3 is indicated with a heatmap. Scale bars: 30 μm. (**B**) Quantification of cell body cCas-3 staining intensity. (**C**) Quantification of synaptic cCas-3 staining intensity. Student’s *t* test was performed for statistical analysis. Data are presented as mean ± SD, *n* = 3; *Z* stacks of 3 sections were analyzed. *P < 0.05.

To investigate whether synaptic degeneration in motor neurons is related to neuronal cell death, we performed ventral nerve cord (VNC) dissections and examined apoptotic marker cleaved Caspase-3 (cCas-3) (19) in the cell body regions and synaptic regions (Figure 6). Control *yw* and Sord-deficient fly VNCs were stained with nucleus marker DAPI, neuronal marker Elav, and cCas-3. We observed a significant increase in synaptic cCas-3 intensity (red-boxed region) and an increasing trend of cell body cCas-3 intensity (yellow-boxed region) in the Sord-deficient flies, indicating increased motor neuron apoptosis. We also examined cCas-3 levels in the brains and found no significant difference between Sord-deficient flies and control (*yw*), either by immunofluorescence or Western blot analysis (Supplemental Figure 2). These results suggest a VNC motor neuron–specific susceptibility to Sord deficiency–induced apoptosis.

### AT-007 improves mitochondrial function in Sord-deficient flies.

We next investigated the molecular and cellular mechanism underlying sorbitol-induced synaptic degeneration. Proper mitochondrial function is critical for maintaining the neuronal function, as neurons have high-energy demands to maintain their membrane potential and survival of distal axons (12). Mitochondrial abnormalities have been implicated in many hereditary neuropathies (10, 11). Compromised mitochondrial function can contribute to peripheral neuropathies by impairing mitochondrial integrity, membrane potential loss, ATP depletion, and subsequent neuronal death (12, 15, 16).

**Figure 7:**
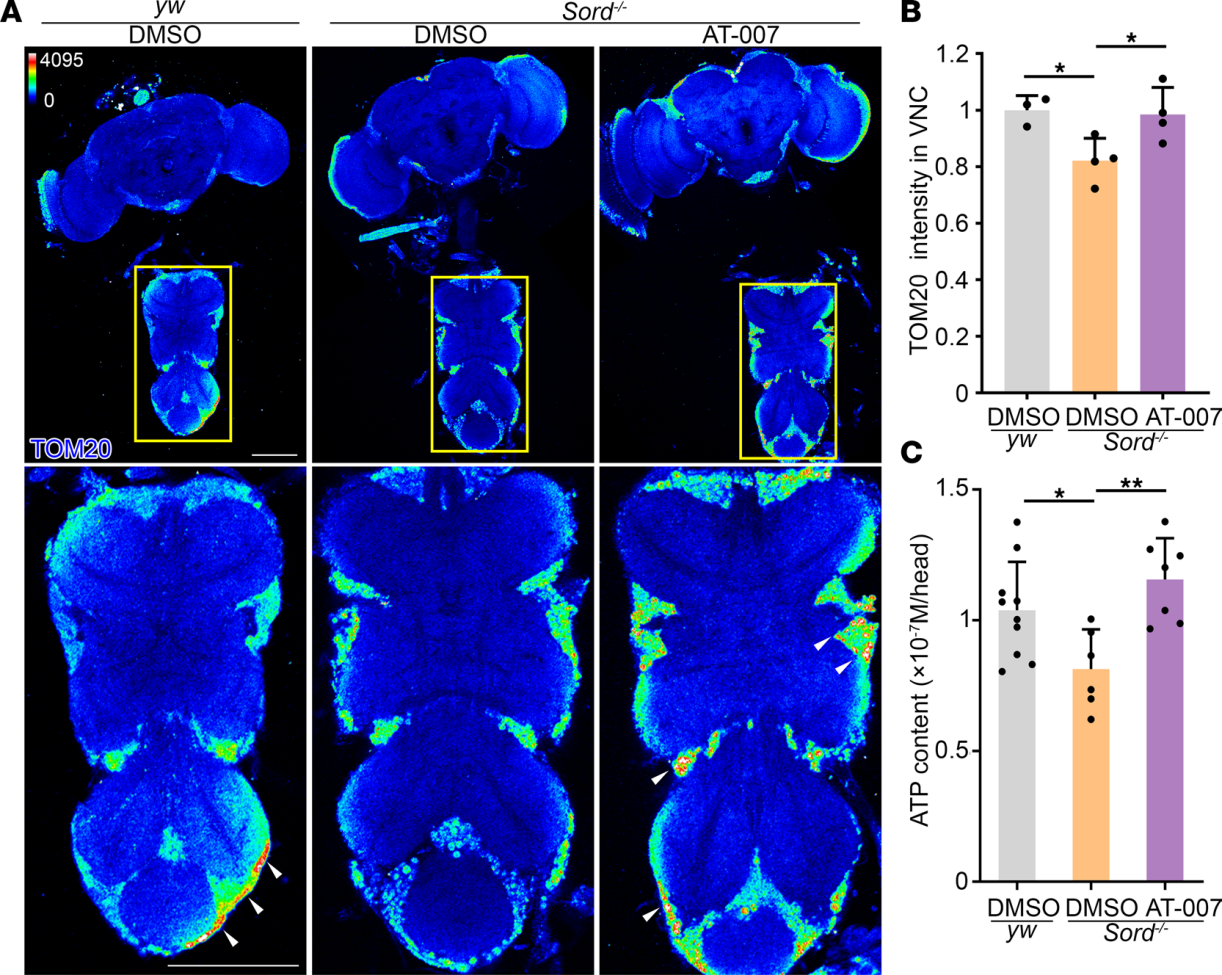
AT-007 improves mitochondrial function in Sord-deficient flies. (**A**) The CNS of the *yw* flies (control) fed with DMSO and Sord-deficient flies fed with DMSO or 10 μg/mL AT-007 at 10 DAE were dissected and stained with TOM20 (heatmap). The VNC areas outlined by the yellow boxes are shown at a higher magnification. White arrowheads indicate TOM20 staining in the motor neuron cell bodies. Scale bars: 100 μm. (**B**) Quantification of TOM20 intensity in the VNC. Data are presented as mean ± SD, *n* = 3–4, *P < 0.05. (**C**) ATP content was measured using a bioluminescence assay from head extractions. Data are presented as mean ± SD *n* = 6–10. *P < 0.05, **P < 0.01. One-way ANOVA was performed for statistical analysis.

To assess mitochondria function, we dissected the fly CNS and stained for translocase of the mitochondrial outer membrane 20 (TOM20), a protein import receptor in the mitochondrial outer membrane (20). In Sord-deficient flies, we observed a decreased level of TOM20 in lower motor neuron cell bodies of the VNC (Figure 7, A and B). To analyze whether sorbitol accumulation impaired mitochondrial function, we performed ATP analysis using a bioluminescence assay (21). We found that ATP content in Sord-deficient fly brains was reduced compared with the control (Figure 7C). Moreover, feeding with AT-007 significantly increased TOM20 expression level in motor neuron cell bodies and ATP content in the brain, indicating improvement of mitochondria function (Figure 7, B and C).

### AT-007 reduces ROS accumulation in Sord-deficient flies and patient-derived fibroblasts.

Mitochondrial dysfunction is commonly accompanied by the accumulation of intracellular ROS (22), primarily generated by NADH/NAD^^+^^ redox imbalance and an oversupply of electron donors to the mitochondrial electron transport chain in oxidative phosphorylation (23). ROS accumulation has been implicated in the etiology of many neuropathies. For example, skin biopsies of patients with Charcot-Marie-Tooth 1A (CMT1A) demonstrate early loss of mitochondrial and antioxidant proteins (24).

**Figure 8:**
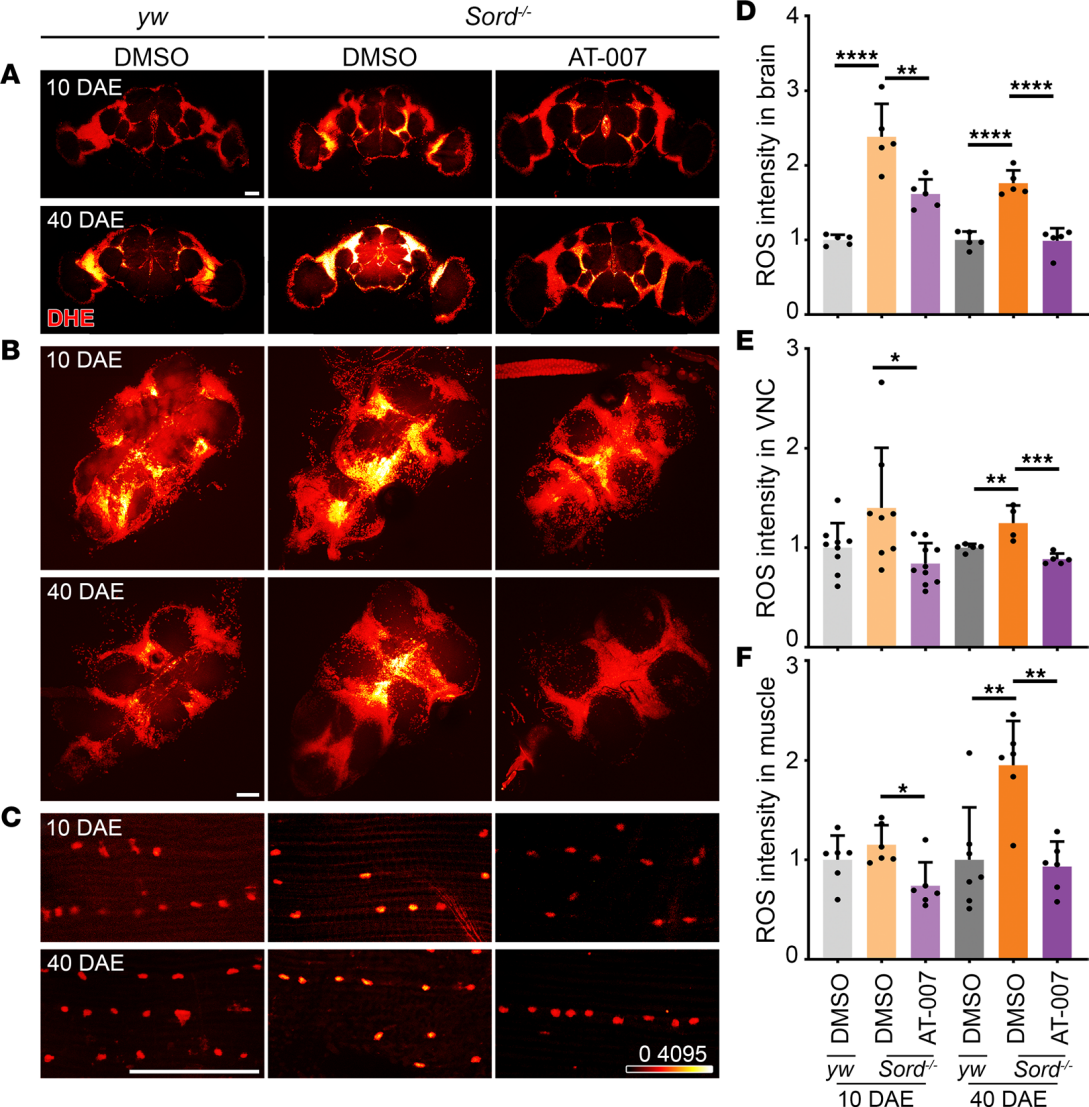
AT-007 reduces ROS accumulation in Sord-deficient flies. (**A**–**C**) Live DHE staining of the brain (**A**), VNC (**B**), and flight muscle (**C**) from *yw* flies (control) fed with DMSO and Sord-deficient flies (*Sord2^MB01265/MB01265^*) fed with DMSO or 10 μg/mL AT-007 at 10 DAE and 40 DAE. The fluorescence intensity of DHE is represented in a heatmap. Scale bars: 100 μm. (**D**–**F**) Quantification of DHE fluorescence intensity in the brain, VNC, and muscle. Data are presented as mean ± SD, *n* = 5–10, *P < 0.05, **P < 0.01, ***P < 0.001, ****P < 0.0001. One-way ANOVA was performed for statistical analysis.

**Figure 9:**
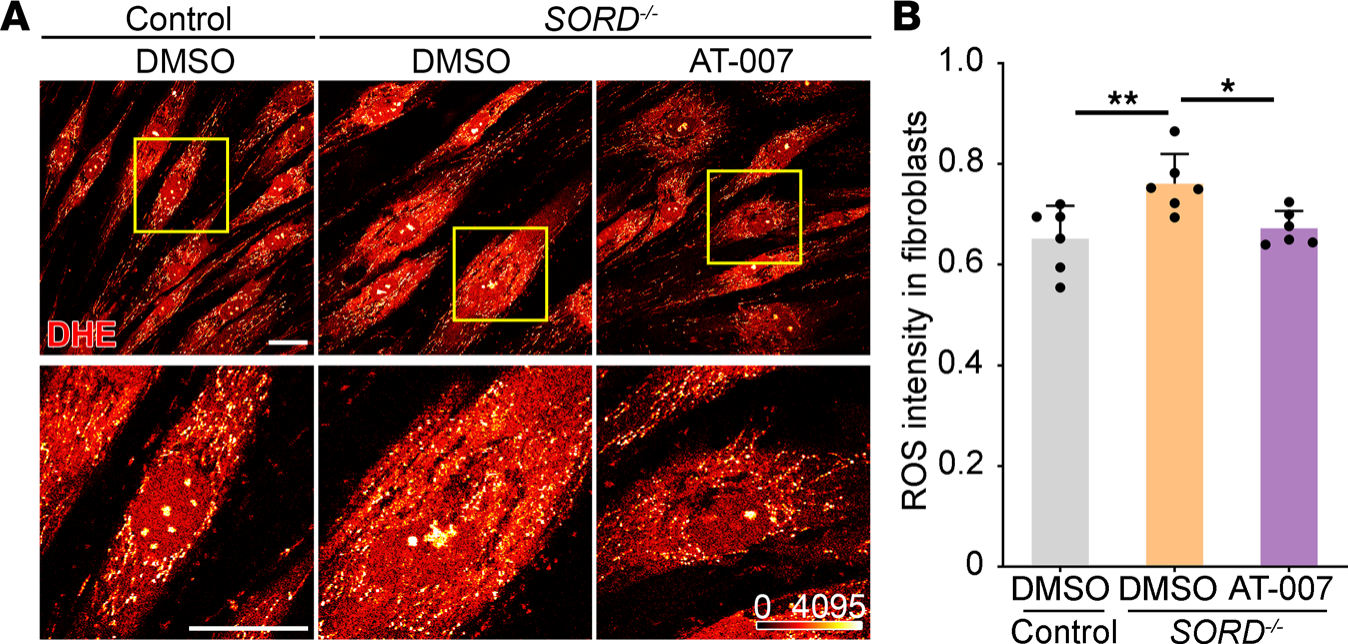
AT-007 reduces ROS accumulation in patient-derived fibroblasts. (**A**) Live DHE staining in patient-derived fibroblasts. A series of 6 random microscopic fields were analyzed, and a representative image was exhibited. Yellow boxed areas are shown in higher magnification to indicate intracellular ROS accumulation. Scale bars: 50 μm. (**B**) Quantification of DHE fluorescence intensity in **A**. Each data point represents the fluorescence intensity of 1 randomly selected field in each image. Data are presented as mean ± SD, *n* = 6, *P < 0.05, **P < 0.01. One-way ANOVA was performed for statistical analysis.

To analyze ROS levels in vivo, we performed live tissue staining with dihydroethidium (DHE), a fluorescent probe for superoxide and hydrogen peroxide species (25). As sorbitol accumulation affected different components of the locomotor system (Figure 3), we examined the fly brains where the upper motor neuron cell bodies reside, the VNC where the lower motor neuron cell bodies reside, and flight muscle fibers (Figure 8). In Sord-deficient flies, we observed an increased ROS level in the brain compared with that in control (*yw*) flies at 10 and 40 DAE. Compared to control flies (*yw*), ROS accumulation in VNC and muscle of Sord-deficient flies was significant at 40 DAE but not at 10 DAE. Compared to DMSO feeding, AT-007 feeding significantly reduced ROS levels in the brain, VNC, and muscle at both early (10 DAE) and late (40 DAE) stages. Consistently, we observed a significant increase in ROS levels in patient-derived fibroblasts, which can be rescued by AT-007 treatment (Figure 9).

## Discussion

SORD deficiency has been shown to cause hereditary neuropathy through elevations in sorbitol level, but the underlying molecular and cellular mechanisms of the disease have not been previously elucidated. Here, we recapitulated the neurodegenerative phenotypes in Sord-deficient flies as described in our previous report (1), including progressive synaptic degeneration and locomotor impairment. Importantly, we demonstrated mitochondrial dysfunction as a critical mechanism underlying SORD deficiency–induced neuropathy, evidenced by increased ROS levels, increased levels of apoptosis, compromised ATP production, and the decreased intensity of mitochondrial marker TOM20. Notably, we reported that a potent and CNS-penetrant next-generation ARI, AT-007, significantly reduced sorbitol levels in patient-derived fibroblasts, iPSC-derived motor neurons, and fly brains. Feeding Sord-deficient flies with AT-007 for 10 days showed a remarkable improvement in locomotor function and restoration of synaptic integrity. AT-007 feeding also reduced ROS accumulation and improved mitochondrial function. Our study highlights the therapeutic potential of AT-007 in treating neuropathies resulting from toxic sorbitol accumulation.

Impaired polyol metabolism and intracellular polyol accumulation have been implicated in many chronic diseases, including diabetic complications, such as diabetic peripheral neuropathy and retinopathy, and metabolic disorders, such as Galactosemia and PMM2-CDG (26, 27). Our recent study and others also identified SORD mutations as the most common genetic cause of autosomal recessive distal hereditary motor neuropathy/CMT2 (dHMN/CMT2) (1, 28). Sorbitol accumulation can result from reduced SORD activity, increased AR activity, or glucose overload. Our present and previous reports observed increased sorbitol levels in both patient-derived fibroblasts and iPSC-derived motor neurons, while patients with SORD mutation showed predominantly neuronal manifestations (1). We also showed that the baseline sorbitol level was significantly higher in motor neurons than in fibroblasts. These findings indicate that neurons are particularly vulnerable to sorbitol toxicity, likely due to their continuous glucose uptake for energy metabolism.

Several mechanisms have been proposed for the cellular toxicity of sorbitol. One proposed mechanism in sorbitol-induced neuropathy is oxidative damage via enhancement of ROS production and/or downregulation of antioxidant defense mechanisms. Our study demonstrated a significant increase in ROS levels in different components of the motor system of Sord-deficient flies, including the brain, the lower motor neuron cell bodies in the VNC, and muscle fibers; it also indicates mitochondrial dysfunction as a consequence of intracellular sorbitol accumulation. First, we show a significant reduction of TOM20 in peripheral motor neuron cell bodies, with concomitant ROS accumulation in the same regions of VNC. Downregulation of key components of mitochondrial import machinery, including TOM20 and translocase of mitochondrial inner membrane 23 (TIM23), are hallmarks of disrupted OXPHOS seen in neurodegenerative disorders such as Parkinson’s disease (29) and Alzheimer’s disease (30). Second, we show decreased ATP production, providing evidence of compromised mitochondrial OXPHOS in Sord-deficient flies. Since most mitochondrial ROS are generated through impaired OXPHOS, our results suggest mitochondrial dysfunction as the primary contributor of oxidative stress in SORD deficiency–induced neuropathy.

Studies on sorbitol-induced diabetic complications in the 1990s favored a hypothesis related to sorbitol-induced hyperosmolarity, resulting in tissue damage. Sorbitol and other polyols cannot easily penetrate membranous structures; therefore, it was hypothesized that their intracellular accumulation increases osmotic pressure and triggers osmotic stress, resulting in cell damage. While intraneuronal accumulation of sorbitol has been associated with impairments of nerve conduction velocities, which are the earliest signs of diabetic neuropathy, the mechanism by which this neuronal damage occurs was never elucidated (31).

Currently, there are no commercially available ARIs in the United States or Europe, and no treatments are yet approved for SORD deficiency. AT-007 from Applied Therapeutics has received Orphan Drug and Pediatric Rare Disease designations from the FDA to treat SORD deficiency, Galactosemia, and phosphomannomutase-2 deficiency, a congenital disorder of glycosylation (PMM2-CDG) featured by increased sorbitol level (32). Preliminary data from the ongoing first therapeutic interventional clinical trial in children aged 2–17 with Classic Galactosemia show that AT-007 significantly reduced toxic galactitol in patients with pediatric Galactosemia and demonstrated a safe and well-tolerated profile (10). A placebo-controlled phase III clinical trial is ongoing to evaluate the efficacy of AT-007 on sorbitol reduction and clinical outcomes of patients with SORD deficiency (NCT05397665). Interestingly, a baseline cross-sectional analysis of patients in the phase III trial demonstrated a statistically significant correlation between blood sorbitol level and lower limb functional metrics, including 10-meter walk-run speed, 4-stair climbing speed, and a sit-to-stand test. Patients with higher sorbitol levels demonstrated worse performance on clinical outcome metrics versus those with lower sorbitol levels of the same age. An interim analysis of the phase III study demonstrated that AT-007 reduced sorbitol levels by a mean of approximately 52% (approximately 16,000 ng/mL) over 90 days of treatment (*P* < 0.001 versus placebo) in patients with SORD deficiency and was safe and well tolerated.

In this study, AT-007 treatment reduced sorbitol levels by approximately 50% in cultured motor neurons and 35% in the *Drosophila* brain and prevented the molecular steps by which sorbitol causes damage to neurons — ROS accumulation, mitochondrial dysfunction, and decreased ATP production. Sorbitol reduction prevented the locomotor phenotype in flies, which parallels the human clinical disease presentation — loss of lower limb function, as measured by lower limb climbing/walking speed. Our findings suggest that sorbitol reduction ameliorates the disease symptoms of SORD deficiency and is sufficient to establish clinical benefit.

Lastly, in our study, Sord-deficient flies were fed AT-007 soon as they eclosed from pupae or at the transition in *Drosophila* lifecycle to adult flies. In SORD-deficient patients, the mean age of onset is 17.2 years, also at the start of adulthood, reflecting similarities in the proposed treatment paradigm (1). This underscores the importance of early diagnosis and treatment in patients with SORD deficiency to prevent potentially irreversible neuronal damage (33).

## Methods

Supplemental Methods are available online with this article.

### Fibroblast cultures

Fibroblasts were obtained by skin biopsy from healthy controls and affected patients homozygous for the *SORD* variant, p.Ala253GlnfsTer27. Fibroblasts were cultured in DMEM, high glucose (4.5 g/L) media supplemented with GlutaMAX, 10% FBS, and ampicillin/streptomycin. Cells were maintained in an incubator at 5% CO__2__ at 37°C. Cells were plated into 10 cm dishes and cultured until reaching 100% cell confluency. Fibroblasts were treated with 100 μM of AT-007 for 96 hours. Cells were supplied daily with fresh media containing AT-007. Fibroblasts were dissociated with trypsin, collected by centrifugation at 100 *g* for 5 minutes at room temperature, and washed with PBS. Cell pellets were resuspended in 300 μL of RIPA buffer (Thermo Fisher Scientific) without a protease inhibitor. Cells were sonicated and spun down at 4°C to remove the remaining cell debris. Protein concentration was measured from cell lysates with the BCA protein detection kit (MilliporeSigma).

### Plasma glucose measurement

A phase I pilot study was conducted to evaluate the safety and pharmacokinetics of AT-007 in patients with SORD deficiency. The study was approved by the Advarra Central IRB committee (no. Pro00055135). Eight patients diagnosed with SORD deficiency were treated with AT-007 (20 mg/kg/day). Plasma glucose level was measured by standard clinical laboratory metrics (ICON Central Laboratories) prior to the first dose, 2 days after the first dose, and after 7 days of consecutive dosing. Data from 7 of 8 patients treated were included (samples from 1 patient were not evaluable).

### iPSC-derived motor neuron cultures

iPSC lines from 3 patients harboring biallelic SORD mutations and 3 healthy controls were plated and grown to 95% confluency before starting differentiation. Differentiation of iPSCs into motor neurons was performed as previously described (34). Following differentiation, motor neurons were sorted out of culture using a CD171-PE antibody (Invitrogen, 12-1719-42) and anti–PE MicroBeads (Miltenyi Biotec, 130-048-801). Motor neurons were plated onto dishes coated with Poly-L Ornithine (10 μg/mL) and Laminin (2.5 μg/mL) and cultured for 4 days before drug treatment. Motor neurons were treated with 100 μM of AT-007 for 3 days and collected for sorbitol analysis.

### Sorbitol measurement in human fibroblasts, motor neurons, and *Drosophila* heads

The sorbitol liquid chromatography–tandem mass spectrometry (LC-MS/MS) assay utilized a protein precipitation extraction with ultra-performance liquid chromatography (UPLC) separation and MS/MS detection to determine sorbitol in human whole blood, using stable labeled internal standard. A 50 μL aliquot of sample was fortified with 10 μL of working internal standard solution (2,500 ng/mL Sorbitol-13C6). To this, 0.400 mL of extraction solvent was added, and the samples were mixed and centrifuged at 2000 *g* for 5 minutes at room temperature. A 0.200 mL aliquot of the supernatant was transferred, and 5 μL were injected into the Sciex API 5000 LC-MS/MS system. The method encompasses a range of 25.0–5,000 ng/mL. The *Drosophila* brain lysate samples were lysed in RIPA buffer solution, diluted 10 times with scrubbed blood, and then analyzed with the above method.

### *Drosophila* stocks and experimental procedures

The following fly strains used in this study were obtained from the Bloomington *Drosophila* Stock Center: *OK371-GAL4*, *yw*, *Sodh2^^MB01265^^, UAS-luciferase RNAi*, *UAS-Sodh1 RNAi*, and *UAS-Sodh2 RNAi*. Flies were reared on cornmeal-molasses-yeast medium at 22°C, 65% humidity, with 12-hour light/12-hour dark cycles. AT-007 was dissolved in DMSO and then mixed into 10 mL fly food at a final concentration of 10 μg/mL. An equal amount of DMSO was mixed into the fly food as a control. The vials were dried at room temperature for 12 hours before feeding.

### *Drosophila* CNS, VNC, and NMJ immunostaining, confocal imaging, and analysis

#### Brain, VNC, and muscle preparations.

Fly brains and VNCs were dissected in phosphate-buffered saline (PBS, pH 7.4) as previously described (35). For *Drosophila* adult flight muscle NMJ dissections, dorsal median muscle groups were carefully isolated under the microscope (36). Samples were fixed in 4% formaldehyde for 10 minutes and washed in PBS containing 0.4% v/v Triton X-100 (PBTX). Samples were then incubated with primary antibodies diluted in 0.4% PBTX with 5% normal goat serum at 4°C overnight, followed by incubation with secondary antibodies diluted in 0.4% PBTX with 5% normal goat serum at 4°C overnight as well as DAPI staining (1:300, Invitrogen, D1306) at room temperature for 10 minutes. The following primary antibodies were used: mouse anti-BRP antibody (nc82, Developmental Studies Hybridoma Bank, NC82, 1:250), rabbit anti-TOM20 antibody (Santa Cruz Biotechnology Inc., sc11415, 1:250), mouse anti-Elav antibody (DSHB, Elav-9F8A9, 1:250), rabbit anti–cCas-3 antibody (Cell Signaling Technology, 9661, 1:250), Cy5-conjugated anti-HRP (Jackson ImmunoResearch, 123-175-021, 1:250), and Alexa Fluor 546–conjugated phalloidin antibody (Invitrogen, A22283, 1:250). The following secondary antibodies were used: Alexa Fluor 555–conjugated anti-mouse secondary antibody (Invitrogen, A21422, 1:250) and Alexa Fluor Cy5–conjugated anti-rabbit secondary antibody (Rockland, 611-110-122, 1:300). The samples were mounted on glass slides with VECTASHIELD Antifade Mounting Medium (Vector Laboratories). Slides were imaged using an Olympus IX81 confocal microscope with 20******×******, 40******×******, or 60******×****** oil immersion objective lens with a scan speed of 8.0 μs per pixel and spatial resolution of 1,024 ******×****** 1,024 pixels. Images were processed using FluoView 10-ASW (Olympus). Quantification was carried out using ImageJ/Fiji (1.53q; NIH).

#### Leg preparation.

Adult hind legs were dissected in PBS, mounted on glass slides with VECTASHIELD, and directly imaged.

### ERG analysis

ERG was performed as previously described (37). Flies were anesthetized with CO__2__ and immobilized on a glass slide. A recording electrode with 3M NaCl was placed on the surface of the left eye, and another reference electrode was inserted into the thorax. After 5 minutes of dark adaptation, flies were given a 1-second light stimulus (Digitimer). The response was amplified unipolarly by GeneClamp 500B (Axon Instruments), and the traces were recorded and analyzed by pCLAMP 10 Electrophysiology Data Acquisition & Analysis Software (ver.10.5).

### *Drosophila* negative geotaxis assay using the AGM platform

#### *Hardware*.

The core of the AGM system consists of a custom-designed acrylic-metal platform that holds 10 plastic vials (ULine, model no. S-21972) and a programmable stepper motor (Trinamic, model no. PD86-3-1278) that, upon receiving a trigger signal, vertically moves the platform 4 times in approximately 3 seconds. Each vial was preloaded with ≤ 7 flies; the geotaxis of each fly was recorded with a digital camera (ImagingSource LLC, model no. DMK23U445). We found that 4 taps of the platform per trial could ensure that most flies were at the bottom of their respective vials and unharmed when recording started (Supplemental Video 3).

#### Hardware control.

A locally written Matlab (Mathworks) computer program commanded the motor to move the platform and instructed the camera to acquire video frames at 30 hertz for 10 seconds once the platform came to rest (Supplemental Video 1).

#### Position tracking.

A custom software was used to detect fly positions in Supplemental Video 2. In brief, our algorithm combined background-subtracted thresholding with constant-velocity Kalman filter prediction and object size discrimination to detect individual fly positions, assign it a tracking number, and update the position with every frame (Supplemental Video 2).

#### Track analysis.

Position information was used to calculate interframe Euclidean distance 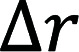 and, from that, average speed over 10 seconds of recording, 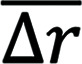/10. Individual fly vertical positions (maximum height, 14 cm) were used to calculate a cohort’s climbing rate. Finally, the difference in SD of horizontal and vertical positions was used to calculate movement direction. Specifically, for a given fly, movement direction = (SD of *y* coordinates – SD of *x* coordinates)/(SD of *y* coordinates + SD of *x* coordinates).

### ATP bioluminescence assay

ATP content in fly heads was measured using an ATP bioluminescence assay (Roche) (21). Briefly, 5 fly heads in each group were homogenized in 200 μL lysis buffer. Samples were boiled for 5 minutes, followed by centrifugation at 18,407 *g* at 4°C for 2 minutes. The supernatant was collected and centrifuged at 18,407 *g* at 4°C for 10 minutes. The supernatant was collected and centrifuged at 18,407 *g* at 4°C for another 2 minutes. Then the supernatant was diluted at 1:20, and 25 μL of the sample was loaded in triplicate in a 96-well plate (triplicate loading). Luciferase activity was measured using a FLUOstar Omega microplate reader (BMG Labtech) with an automated injection system. An aliquot of 25 μL of luciferase reagent was injected, and the luminescence signal was monitored. An ATP standard curve was established to determine the ATP concentration.

### DHE staining

ROS levels were assessed using live staining DHE, a fluorescent probe for superoxide and hydrogen peroxide.

#### DHE staining in Drosophila.

Fly brains, VNCs, and muscles were dissected in warm Schneider’s media. The samples were then incubated in 30 μM DHE for 15 minutes. The samples were washed with PBS 3 times (5 minutes each), and a final wash with PBTX for 5 minutes. The samples were mounted on glass slides with VECTASHIELD Antifade Mounting Medium. Brain slides were immediately imaged using an Olympus IX81 confocal microscope with a 20******×****** (brain), 40******×****** (VNC), or 100******×****** (muscle) oil immersion objective lens with a scan speed of 8.0 μs per pixel and a spatial resolution of 1,024 ******×****** 1,024 pixels (brain and VNC) or a scan speed of 10.0 μs per pixel and a spatial resolution of 2,048 ******×****** 2,048 pixels (muscle). All images were processed using FluoView 10-ASW (Olympus). ROS intensity was quantified using ImageJ/Fiji (1.53q).

#### DHE staining in fibroblasts.

Fibroblasts were cultured in DMEM (Corning, 15-013-CV) supplemented with 10% FBS (ATCC, 30-2020) at 37°C with 5% CO__2__ in the VWR symphony incubator. The cells were seeded in live cell image dishes (SPL Life Sciences, 200350) with 50% confluence for 24 hours. Cells were treated with DMSO 0.1% or 100 μM of AT-007 for 96 hours. Cells were washed with fresh medium and treated with 5 μM of DHE (Thermo Fisher Scientific, D11347) and 75 nM of MitoTracker (Thermo Fisher Scientific, M7514, as a control) in 200 μL of fresh medium for 15 minutes. Then, the cells were washed with prewarmed PBS. In total, 200 μL of prewarmed fresh medium was added to the dish, and the cells were imaged immediately. Images were processed using Olympus FluoView 10-ASW and analyzed using ImageJ.

### Statistics

Biological sample size (*n*) and *P* values are indicated in the corresponding figure legends. One-way ANOVA with Bonferroni’s post hoc test was applied to compare multiple groups, and 2-tailed Student’s *t* test was applied to compare between 2 groups. *P* < 0.05 was considered statistically significant. All statistical analyses were performed in GraphPad Prism software (version 7.04).

## Author contributions

YZ (co–first author), S. Shendelman, SZ, and RGZ conceptualized the study. YZ, AGL (co–first author), S. Syed, S. Shendelman, SZ, and RGZ designed the study’s methodology. YZ, AGL, APR, TC, NOV, XT, CY, M. Saporta, M. Shy, SZ, and RGZ conducted the investigation. YZ, AGL, and RGZ created the visualizations. S. Shendelman, SZ, and RGZ acquired funding for the study. RGZ administered and supervised the project. YZ, AGL, and RGZ wrote the original draft. YZ, AGL, APR, TC, NOV, XT, S. Syed, CY, RP, and S. Shendelman reviewed and edited the manuscript. Co-first authorship order was decided based on the timeline of contributions.

## Supplementary Material

Supplemental data

Supplemental video 1

Supplemental video 2

Supplemental video 3

## Version 1 04/04/2023

In-Press Preview

## Version 2 05/22/2023

Electronic publication
