# Supplementary material for: Sorbitol reduction via govorestat ameliorates synaptic dysfunction and neurodegeneration in sorbitol dehydrogenase deficiency
Source: JCI Insight. 2023 May 22;8(10):e164954. doi: 10.1172/jci.insight.164954 (PMC10322690; doi:10.1172/jci.insight.164954)
Supplement: Supplemental data [file jciinsight-8-164954-s205.pdf]

Sorbitol reduction via govorestat ameliorates synaptic dysfunction and neurodegeneration in sorbitol dehydrogenase deficiency

Yi Zhu[1*], Amanda G. Lobato[1,2*], Adriana P. Rebelo[3], Tijana Canic[4], Natalie Ortiz-Vega[1,5], Xianzun Tao[1], Sheyum Syed[4], Christopher Yanick[6,7], Mario Saporta[6], Michael Shy[8], Riccardo Perfetti[9], Shoshana Shendelman[9], Stephan Züchner[3], R. Grace Zhai[1#]

[1]Department of Molecular and Cellular Pharmacology, University of Miami Miller School of Medicine, Miami, FL, USA

[2]Graduate Program in Human Genetics and Genomics, University of Miami Miller School of Medicine, Miami, FL, USA

[3]Dr. John T. Macdonald Foundation Department of Human Genetics and John P. Hussman Institute for Human Genomics, University of Miami Miller School of Medicine, Miami, FL, USA

[4]Department of Physics, University of Miami, Coral Gables, FL, USA

[5]Graduate Program in Cellular and Molecular Pharmacology, University of Miami Miller School of Medicine, Miami, FL

[6]Department of Neurology, University of Miami Miller School of Medicine, Miami, FL, USA

[7]Graduate Program in Neuroscience, University of Miami Miller School of Medicine, Miami, FL, USA

[8]Department of Neurology, Carver College of Medicine, University of Iowa, Iowa City, Iowa, USA

[9]Rsch & Development, Applied Therapeutics, New York, NY, USA

*Co-first authors

#Correspondence: gzhai@med.miami.edu

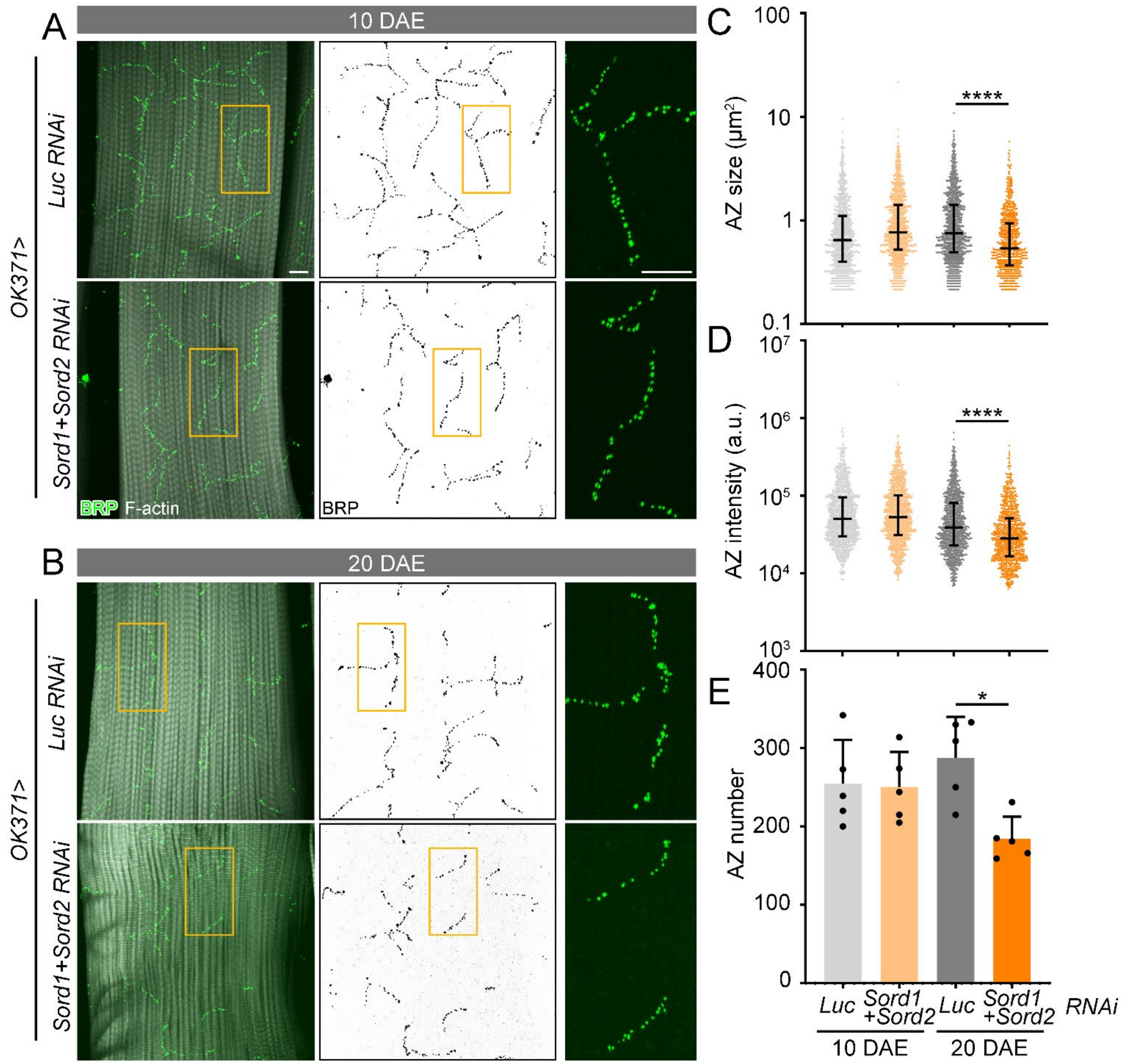

Supplementary Figure S1. Motor neuron-specific knockdown of Sord1 and Sord2 leads to AZ structural abnormalities in the flight muscle NMJs. (**A, B**) Flight muscles of flies with motor neuron-specific knockdown of luciferase (control) or Sord1+Sord2 were dissected at 10 and 20 DAE and stained with BRP, HRP, and phalloidin (F-actin). Boxed areas are shown in higher magnification. Scale bars: 10 μm. (**C-E**) Quantification of AZ size, intensity, and number in flight muscle NMJs. Data are presented as median ± interquartile range (**C, D**) and mean ± SD (**E**). *$p < 0.05$, ****$p < 0.0001$. n=5. Student's t-test was performed for statistical analysis.

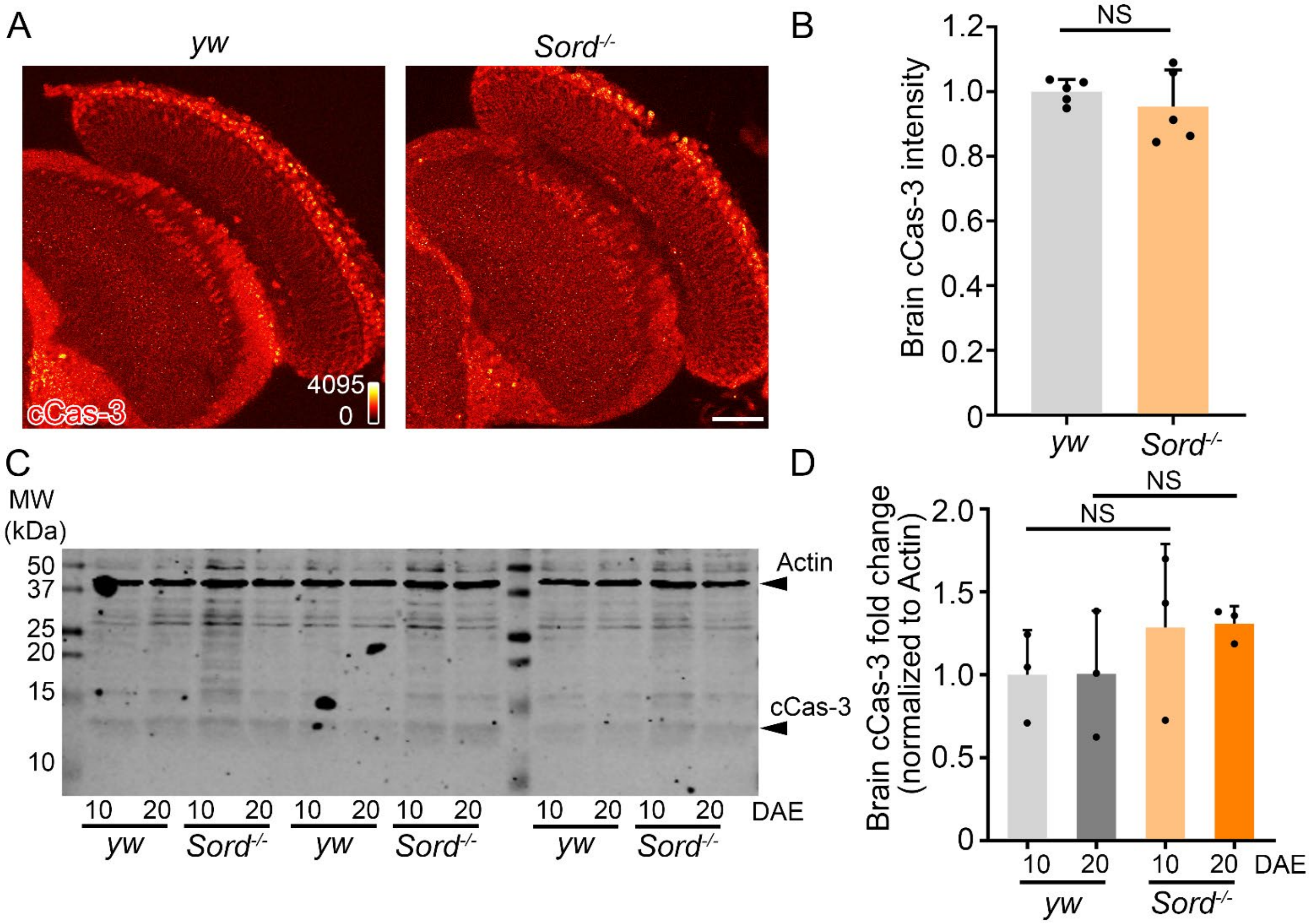

Supplementary Figure S2. Cleaved caspase-3 (cCas-3) in the brain shows no difference between *yw* and Sord-deficient flies. **(A)** 10 DAE *yw* (control) and Sord-deficient flies were dissected and stained for cCas-3. Scale bar: 30µm. **(B)** Quantification of cCas-3 in the brain. n = 4 per genotype. Data are presented as mean ± SD. **(C)** Western blot analysis of cCas-3 staining for *yw* (control) and Sord-deficient fly heads at 10 and 20 DAE n = 3. Actin is used as a loading control. (**D**) Quantification of cCas-3 staining normalized to Actin. Student's t-test was performed for statistical analysis. NS: not significant.

Supplementary Methods:

Western blot analysis. For analyzing cCas-3 expression (Supplemental Figure 2C), 10 heads of each genotype were homogenized using RIPA (radioimmunoprecipitation assay) buffer. Extracted samples were mixed with Laemmli sample buffer (2% SDS, 10% glycerol, 62.5mM Tri-HCl, 0.001% bromophenol blue, and 5% β-mercaptoethanol) and denatured at 95°C for 10 minutes. Proteins were separated by SDS-PAGE (sodium dodecyl sulfate-polyacrylamide gel electrophoresis) and transferred to a nitrocellulose membrane. After blocking at RT for 1 hour, the membrane was incubated with primary antibody at 4°C overnight, followed by secondary antibody incubation for 1 hour at RT. The membrane was scanned by an Odyssey Infrared Imaging system (LI-COR Biosciences) and images were analyzed using Image Studio (version 4.0). The primary antibody dilution for anti-cCas-3 was 1:1000 (Cell Signaling Ca# 9661), and β-actin was 1:5,000 (Sigma-Aldrich Ca# A1978). The secondary antibody dilution for DyLight 680 conjugated anti-Rabbit IgG was 1:10,000 (Rockland Ca# 611-144-002), and DyLight 800 conjugated anti-Mouse IgG was 1:10,000 (Rockland Ca# 610-145-002).
